# 

**Published:** 1892-12-10

**Authors:** 


**Notices of Births, Deaths, and Marriages,** are inserted in this column at a charge of 2s. 6d. for an announcement not exceeding four lines.

MARRIAGES.

Churchill—Gee.—December 6th, at the Church of the Ascension, Blackheath, Alick Beaumont Churchill, of Long Buckby, Northamptonshire, to Millicent Dorothy Gee, only daughter of Robert Willett Gee, Esq., of Methwold, Norfolk.

**Notice to Advertisers and Subscribers.**

All Advertisements, Orders for Copies of The Hospital, and Business Communications should be addressed to The Publisher, *not to the Editor*, The Hospital, 140, Strand, W.C.

The Cost of Subscription, post free, is as follows:—Per week, 2½d.; per quarter, 2s. 6d.; per half-year, 5s.; per annum, 8s. 6d. Foreign:—Per annum, 10s. 8d.; payable, in all cases, in advance.

**"Burdett's Hospital Annual," 1892—1893.**

Fourth year of publication. 700 pp. Boards, gilt lettering. A most complete guide to British and Colonial Hospitals, Dispensaries, Nursing and Convalescent Institutions. and Asylums. Price 5s. Published by The Scientific Press (Limited), 140, Strand, W.C.

**NOTICE.—The Index for Vol. XII. (ending September, 1892) is on sale at the Office of this Journal, price 2½d., post free.**

### Books Received.

Stanford.

"Mediteranean Winter Resorts." By Reynolds Ball. Second Edition.

Ward, Lock, Bowden, and Co.

"The Biographical History of Guy's Hospital." By Samuel Wilks, M.D., and G. T. Bettany, M.A.

**Periodicals and Pamphlets.**

Religious Tract Society.—Starshine, the Christmas Number of the Girl's Own Paper, the Christmas Number of the Boy's Own Paper, The Sunday at Home, Girl's Own Paper, Boy's Own Paper, The Leisure Hour.

**Other Publications.**

The Young Gentlewoman; Edinburgh Royal Infirmary Pharmacopœia; Review of Reviews; Lett's Medical Diary for 1893; The Toilers of the Deep; The Scalpel; The Clinical Journal.

Annual Reports.—Stamford Nursing Society, John Murray's Asylum.

### SCRAPS AND GLEANINGS.

Twelve cases of small-pox were reported last week to the Medical Officer of Health for Islington.

A large Kosmic Fête is to take place in Dublin in May, 1893, in aid of the building fund of the City of Dublin Hospital.

Mrs. Fearnsides has sent a further donation of £250 to the Irish Distressed Ladies' Fund, making a total of £1,000.

A Working Committee has just been elected to carry on the necessary details connected with the building of the new Southport Infirmary.

The return of the Registrar-General for Scotland states that in the country during the three months ending with September 30,424 births, 15,622 deaths, and 6,684 marriages were registered.

Prince Henry of Battenberg has promised to preside at the forty-eighth anniversary dinner of the German Hospital, which will take place at the Hotel Métropole on Wednesday, May 10th, 1893.

The annual court of the Seamen's Hospital is announced to be held on February 1st, 1893. The Lord Mayor has consented to preside, and the meeting will be at the Mansion House at three o'clock.

Dr. Whiting, who attended the late Lord Tennyson for many years when the Poet Laureate was at Blackdown, died suddenly on Monday 21st inst., from apoplexy, aged 63. Dr. Whiting was a personal friend of Lord Tennyson's, as well as his medical attendant.

The late Mr. George Trimmer, of Farnham, a member of the Surrey County Council, has left £15,000 for the endowment of a cottage hospital for Farnham, £9,000 for the erection of almshouses there, and £500 to the Royal Surrey County Hospital at Guildford.

At a recent meeting of the Royal Commission on Vaccination, Sir James Paget in the chair, Dr. McVail, of Glasgow, spoke at considerable length in favour of compulsory vaccination, proving by statistical returns the value of inoculation as a preventive of small-pox.

An epidemic of typhus fever has broken out in Dundee. The sanitary authorities propose to use the infirmary at the Barracks for isolating purposes. The building consists of two storeys, and contains accommodation for 100, exclusive of nurses and other attendants.

At the quarterly court of the Governors of the Hospital for Consumption, Brompton, legacies amounting to nearly £5,000 were announced; also a grant of £1,458 from the Hospital Sunday Fund to the charity. The Prince of Wales has sent a present of wine for the use of the patients.

The Model Village Hospital and Institute erected at Almondsbury by Mr. Shotto Vere Hare. as a memorial to his wife, was opened by the Duchess of Rutland. Mr. Ponting is the architect. The hospital is not only a very handsome building, but is fitted up with every modern improvement and requirement.

Lord Derby has made a free gift of the land for the site of the proposed Ormskirk Cottage Hospital. It is the intention of the Committee to make provision for six beds first (two wards, three beds in each), as well as two small wards for special purposes, with every accommodation for the medical staff and nurses.

The Treasurer of the British Home for Incurables has received from Mr. Pinder Simpson the sum of £250, being a donation sent by him from the funds placed at his disposal as executor of the late Mrs. Eliza Holm. (and to be applied to the building fund of the new Home at Streatham, now in course of erection.

Many improvements have been made during the year in the wards and throughout the building of the Stirling Royal Infirmary. The report states the number of in-patients to have been 231 and out-patients 1,734. The income amounted to £1,002 9s. 4d., and the expenditure to £1,068 13s., leaving a deficiency of £66 3s. 8d.

The Duke of York has graciously consented to preside at a festival dinner, which is to take place early next spring in aid of the funds for the completion of the buildings of the Great Northern Hospital, Holloway Road. Towards the £27,000 required for the cost of the extension, with furniture and fittings, only £8,000 has been promised.

A two-days' sale of fancy work in aid of the extension fund of the Children's Hospital, Paddington Green, was held at Lord Brassey's house, Park Lane, on November 30th and December 1st. Short concerts, under the direction of Mr. de Barri Crawshay, were given at intervals, and Lord Brassey's Indian Museum was thrown open to the visitors.

During the past twelve years 1,200 patients have been received into the Bolingbroke Hospital for paying patients. At a meeting held on November 11th, presided over by Lord Battersea, it was agreed to raise at once a fund, if possible, of £2,000 to prevent the closing of so useful an institution, which unfortunately at the present time is in financial straits.

At the annual meeting of the Portsmouth Eye and Ear Infirmary, the Mayor said that the new out patients' department had been completed, thanks largely to an anonymous gift of £1 000, but there was still a debt on the building. The balance-sheet showed the expenditure to have been £297 1s. 6d., and the receipts £247 13s. 10d., leaving a deficiency of £49 7s. 8d.

The twenty-sixth annual report of the Reigate and Redhill Cottage Hospital shows that 186 cases came under treatment during the year. The average duration of each case was 20 days. Certain necessary sanitary and other works in connection with the hospital have been carried out, the expense being defrayed by further donations from subscribers.

Her Royal Highness Princess Mary Adelaide of Teck opened a bazaar in aid of the Enfield Cottage Hospital, on Saturday, the 19th inst. The bazaar was organized with a view to defray the expenses incurred in adding a new ward containing three beds and the necessary nurses' apartments in commemoration of the Jubilee year. It is 300 years since Enfield received a visit from Royalty.

The Governors of Addenbroke's Hospital, Cambridgeshire, have decided, upon the advice of Mr. Rogers Field, to reconstruct the entire drainage system of the hospital. This will cost between £2,000 and £3,000, and as they have not the necessary funds to carry out this work, the Committee makes a special appeal to the public for assistance, in order that they may not be obliged, otherwise, to realise some of the invested capital and curtail the income.

### The Student of Medicine.

[All communications for this Supplement should be addressed to The Editor of The Hospital, at 140, Strand, with the words, "Students' Column," written in the left hand top corner of the envelope.]

### APPOINTMENTS.

Bailey, Samuel H., M.D., M.B., C.M.Aberd., appointed Medical Officer for the Fifth District of the Nottingham Union. Ballantyne, J. W., M.D.Edin., appointed Obstetric Physician and Gynæcologist to the Western Infirmary, Edinburgh. Brown, Murdock, M.B., C.M.Edin., appointed Consulting Physician to the Western Infirmary, Edinburgh. Bury, R. F., M.R.C.S., L.R.C.P., appointed House Surgeon to St. George's Hospital. Charlton, F. J., L.R.C.P.Lond., M.R.C.S., appointed Medical Officer for the Fourth Sanitary District of the Wimborne Union. Cosens, W. B., L.R.C.P.Lond., M.R.C.S.Eng., appointed Honorary Surgeon to the Taunton and Somerset Hospital. Dalison, B. E., M.B., C.M.Edin., appointed Medical Officer for the Puddletown District of the Dorchester Union. Donald, James, M.R.C.S., re-appointed Medical Officer of Health for Ham. Dougan, William, M.D.Glas., appointed Medical Officer to the Glasgow Post Office. Evans, J. J., M.B., C.M.Edin., appointed Resident Medical Officer to the Carmarthenshire Infirmary. Fleming, R. A., M.A., M.B., C.M.Edin., appointed Senior Physician to the Western Infirmary, Edinburgh. Gardner, Mr. Harold, appointed House Surgeon to Charing Cross Hospital. Garrod, A. E., M.A., M.D., appointed Assistant Physician to the Hospital for Sick Children, Great Ormond Street. Gunn, Donald, F.R.C.S., appointed Ophthalmic Surgeon to the Hospital for Sick Children, Great Ormond Street. Hall, Alfred, M.R.C.S., re-appointed Medical Officer for the Butterton and Grindon Sanitary District of the Leek Union. Hallowes, A. H. B., M.R.C.S. Eng., L.S.A., appointed Medical Officer for the No. 2 District of the Maidstone Union. Haycock, H. E., L.R.C.P.Edin., M.R.C.S. Eng., appointed Medical Officer for the Thirteenth District of the Basford Union. Hopkins, G. Herbert, L.R.C.P.Lond., M.R.C.S., appointed Senior Surgeon to the Swansea General Hospital. Hoskins, Dr. W., appointed Medical Officer for the Nazeing District of the Epping Union. Hughes, E. Lucas, M.R.C.S., L.R.C.P.Lond., appointed Physician's and Surgeon's Assistant to the Out-patients at University College Hospital. Knowles, E., M.D., C.M.Aberd., appointed Public Vaccinator for the Birkenhead District of the Birkenhead Union. Leggatt, Gerrard S., M.R.C.S.Eng., appointed Medical Officer for the Out-door Poor and the Workhouse of the Hoo Union. Lloyd, T. F., M.R.C.S.Eng., L.S.A., re-appointed Medical Officer for the No. 4 District of the Reigate Union. Marshall, Dr., appointed Medical Officer to the Bo'ness Parochial Board. Muir, Robert Douglas, M.R.C.S.Eng., L.R.C.P.Lond., appointed House Physician to Charing Cross Hospital. Neville, Thomas, M.D., Ch.Irel., appointed Divisional Surgeon to the Police attached to the Gerald Road Police Station in the B Division, S.W. Prosser, Frank, M.B., C.M.Glasg., appointed Medical Officer of Health for the Urban Sanitary District of the Prescot Union. Ransome, H. F., L.R.C.P., M.R.C.S., appointed Honorary Medical Officer to the Manchester Hospital for Consumption and Diseases of the Throat. Reece, James, M.D., D.P.H., appointed Medical Officer of Health to the Kingstown Town Council. Richardson, R. T., M.R.C.S., appointed Medical Officer for the North Bradley and Southwick Sanitary District of the Westbury Union. Ryan, Dr. A. B., appointed Medical Officer of the Silvermines Dispensary, District of the Nenagh Union.

### VACANCIES.

The following vacancies are announced this week, the amount of salary in each case being given after the name of the institution:

### Resident Medical Officers.

Belgrave Hospital for Children, 79, Gloucester Street, S.W. House Surgeon. Board, lodging, fuel, and light found. Applications, endorsed on envelope "House Surgeon," to the Honorary Secretary, by December 16th.

Brecon Infirmary. House Surgeon (see advertisement below).

Brighton, Hove, and Preston Dispensary, House Surgeon to the Western Branch, unmarried, doubly qualified—Salary £140 per annum (less board at 8s. per week), with furnished apartments, coal, gas, and attendance. Applications to the Assistant Secretary by December 15th.

ASSISTANT HOUSE SURGEON.

**THE STUDENT OF MEDICINE—(*continued*).**

General Hospital, Birmingham. Assistant House Surgeon, surgical qualification required. Board, lodging, and washing provided. Applications must be received by December 31st. Election on January 6th, duties commence January 15th.

Fisherton Asylum, Salisbury. Clinical Assistant for six months. Board, lodging, and washing found. Applications to Dr. Finch at the Asylum.

Killarney District Lunatic Asylum. Assistant Medical Officer. Salary, £100 per annum, with allowances valued at £100 yearly. Candidates must be unmarried and not over 30. Applications to Dr. L. T. Griffin, Resident Medical Superintendent. Election on January 19th.

Northumberland County Lunatic Asylum, Morpeth. Assistant Medical Officer, unmarried—Salary £120 per annum, increasing £10 yearly to £150, with furnished apartments, and board and lodging. Applications to Dr. McDowall at the Asylum by December 15th.

Parochial Board of Edinburgh. Resident Medical Officer for the Poorhouse, Craiglockhart. Salary £80 per annum, with board and lodging. Applications to G. Greig, Inspector, 2, Forrest Road, Edinburgh, by December 10th.

Royal Cornwall Infirmary, Truro. House Surgeon, unmarried, doubly qualified. Salary £150 per annum, with furnished apartments, fire, light, and attendance. Applications to the Secretary before December 14th.

Royal London Ophthalmic Hospital. Senior House Surgeon (see advertisement on previous page).

St. Thomas's Hospital Medical School, S.E. Resident Assistant Physician, a Resident Assistant Surgeon, three Demonstrators of Anatomy, Demonstrator of Physiology, two Hospital Registrars, Obstetric Tutor and Registrar, eight House Physicians, eight House Surgeons, and eight Assistant House Surgeons, eight Obstetric House Physicians, four Ophthalmic House Surgeons, Clinical Assistants in the Special Departments, Clinical Clerks and Dressers in the Wards and in the Out-patient Departments. Obstetric Clerks, Assistants to the Teachers of Practical Surgery and of Materia Medica, Assistants in the Physiological and Pathological Laboratories, Anatomical Registrars and Prosectors. Applications to G. H. Makins, Dean.

Westminster General Dispensary, 9, Gerrard Street, Soho, Resident Medical Officer. Appointment for one year. Applications to J. J. Johnson, Secretary, by December 15th.

**Non-Resident Medical Officers.**

Owens College, Manchester. Junior Demonstrator in Physiology and Histology. Salary £100 per annum. Applications to the Registrar by December 12th.

Rathdrum Union, Arklow Dispensary. Medical Officer. Salary £110 per annum, and fees. Applications to James Hannagen, Honorary Secretary, Curranstown, Arklow. Election on December 12th.

St. Andrews University, Dundee. Demonstrator of Anatomy. Salary, £120 per annum. Applications to the Secretary by December 17th.

